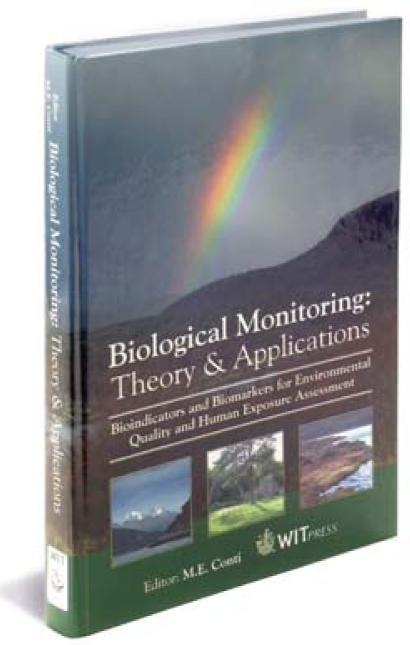# Biological Monitoring: Theory and Applications—Bioindicators and Biomarkers for Environmental Quality and Human Exposure Assessment

**Published:** 2008-07

**Authors:** Dana B. Barr

**Affiliations:** Dana Barr has been a research chemist at the Centers for Disease Control and Prevention for 21 years. Her research interests include analytical methods development and biological monitoring of human exposure to environmental toxicants, with a special emphasis on child health. She has authored or coauthored over 180 publications related to her research interests

*Biological Monitoring* is a newly published book whose chief aim is to provide an overview on the current knowledge of biological monitoring by evaluating the quality of ecosystems and human health. The book, edited and mostly written by Marcelo Enrique Conti, is composed of seven relatively dense chapters that deal mostly with monitoring ecosystem changes that result from environmental insults.

The first and second chapters set the foundation for the subsequent chapters by introducing the book’s central theme—that biota in the ecosystem react or respond to environmental stressors, and thus can serve as sensitive bioindicators of environmental quality. The next two chapters discuss aquatic environments, both freshwater and marine environments, and bioindicator assessments of water quality. The authors discuss the various advantages and disadvantages of using invertebrates, fish fauna, and plant systems as indicators of water quality parameters such as nutrient load, presence of heavy metals and other toxicants, and pH. They also introduce the concept of the Index of Biotic Integrity, which characterizes the ability of an environment to remain balanced and integrated into the specified ecosystem.

The fifth chapter delves into the use of lichens as bioindicators of air quality. A lichen is defined as the product of a symbiotic relationship between a fungus and an alga, with the alga supplying the nutrients in the form of chlorophyll and the fungus supplying water and minerals. If either the production of nutrients or the water is altered, the lichen can show changes in their components such as chlorophyll content or respiration level. Because environmental air factors (e.g., ozone content, fluoride and chloride content, radionuclide concentrations) can modulate these specific lichen parameters, they are considered good bioindicators of air pollution. In addition, as with other bioindicators, direct measurements of specific environmental pollutants such as heavy metals can be made in lichens. Detailed information on two methods for the calculation of the Index of Atmospheric Purity (IAP; a quantitative evaluation of the level of air pollution) is given. Lower IAP values correspond to higher levels of air pollution.

The chapter on human biomonitoring follows, with particular emphasis on toxicants such as metals and persistent organic pollutants that have been well documented in the literature over the last several decades. Biomonitoring of exposure, susceptibility, and effect were evaluated for each pollutant listed. This chapter provides a rather superficial, outdated overview of the state of human biomonitoring. It does not even mention current issues in human biomonitoring such as challenges in interpreting biomonitoring data and the current ability of analytical instrumentation to detect contaminants, their metabolites, or reaction products at levels with unknown clinical relevance.

The last chapter is somewhat confusing, offering various multivariate approaches that could potentially be applied to ecosystems biomonitoring studies. This chapter explores the use of unsupervised and supervised pattern recognition techniques including Hotelling’s *T*^2^ test, discriminant analysis by Mahalanobis distance, and linear discriminant analysis. Because this material is mentioned only perfunctorily, it serves as a distraction from the main objectives of the book, rather than an enhancement of the material.

Although well written and somewhat informative, the book did not provide information I had hoped to glean from the it. Perhaps because of the title, my expectation from the book was to learn more about human biological monitoring, but the focus was more toward ecosystems monitoring. The references, for the most part, were citations of the editor’s or author’s own work, and those that were not self citations were mostly dated (> 5 years old). Although somewhat lacking in objectivity and omitting some more state-of-the-science research, this book does provide a nice overview of the current science in ecosystems monitoring and may serve as a suitable textbook for individuals just entering the field who need to delve deep into the theory and applications of this field.

## Figures and Tables

**Figure:**